# Value of Cardiopulmonary Exercise Testing in Prognostic Assessment of Patients with Interstitial Lung Diseases

**DOI:** 10.3390/jcm11061609

**Published:** 2022-03-14

**Authors:** Beate Stubbe, Till Ittermann, Anita Grieger, Charlotte Walther, Sven Gläser, Ralf Ewert

**Affiliations:** 1 Internal Medicine B, Pulmonary Medicine, University Medicine Greifswald, Ferdinand-Sauerbruchstrasse, 17475 Greifswald, Germany; anita_grieger@gmx.de (A.G.); lotte.walther@web.de (C.W.); ralf.ewert@med.uni-greifswald.de (R.E.); 2 Institute for Community Medicine, SHIP, University Medicine Greifswald, 17475 Greifswald, Germany; till.ittermann@med.uni-greifswald.de; 3 Department of Pulmonary Medicine, Vivantes Hospital Berlin, 12351 Berlin, Germany; sven.glaeser@vivantes.de

**Keywords:** interstitial lung disease, idiopathic pulmonary fibrosis, comorbidities, lung function, cardiopulmonary exercise testing, prognosis, all-cause mortality

## Abstract

Background: Interstitial lung disease (ILD) is associated with high rates of comorbidities and non-infectious lung disease mortality. Against this background, we aimed to evaluate the prognostic capacity of lung function and cardiopulmonary exercise testing (CPET) in patients with ILD. Materials and Methods: A total of 183 patients with diverse ILD entities were included in this monocentric analysis. Prediction models were determined using Cox regression models with age, sex, body mass index (BMI), and all parameters from pulmonary function testing and CPET. Kaplan–Meier curves were plotted for selected variables. Results: The median follow-up period was 3.0 ± 2.5 years. Arterial hypertension (57%) and pulmonary hypertension (38%) were the leading comorbidities. The Charlson comorbidity index score was 2 ± 2 points. The 3-year and 5-year survival rates were 68% and 50%, respectively. VO_2_peak (mL/kg/min or %pred.) was identified as a significant prognostic parameter in patients with ILD. The cut-off value for discriminating mortality was 61%. Conclusion: The present analyses consistently revealed the high prognostic power of VO_2_peak %pred. and other parameters evaluating breathing efficacy (VÉ/VCO_2_ @AT und VÉ/VCO_2_ slope) in ILD patients. VO_2_peak %pred., in contrast to the established prognostic values FVC %pred., DLCO/KCO %pred., and GAP, showed an even higher prognostic ability in all statistical models.

## 1. Introduction

The main entities of interstitial lung diseases (ILDs) are idiopathic pulmonary fibrosis (IPF), idiopathic nonspecific interstitial pneumonia (NSIP), sarcoidosis with fibrosis, and hypersensitivity pneumonitis [1]. Some revisions of ILD entities have been performed recently [1,2]. Due to the diagnostic complexity of ILDs, a dynamic integrated approach using multidisciplinary discussion (MDD) is considered the standard for classification of these diseases [3]. ILDs (sarcoidosis included) occupy the third position among non-infectious pulmonary diseases in terms of the mortality rate [4]. The German INSIGHT-IPF-Registry data indicated a 1-year and 2-year survival rate of 87% versus 46% and 62% versus 21%, respectively, for patients with versus without antifibrotic therapy [5]. In comparison to the general population, patients with ILDs more frequently show several comorbidities, especially cardiac diseases, diabetes mellitus, dyslipidaemia, obstructive sleep apnoea, cancer, and depression [6,7,8,9]. Additionally, these diseases are associated with limited cardiorespiratory fitness (CRF). The CRF, which is measured as ‘power of work’ [10] or ‘maximum oxygen uptake (VO_2_peak)’ [11] in addition to other parameters [12,13], has a prognostic influence in ILD subgroups. There are multiple pathophysiologic reasons for the reduced CRF, such as lung functional pathologies, disturbances in gas exchange, and haemodynamic limitations [14]. Therefore, it seems plausible that pulmonary hypertension (PH), which indicates an impairment of pulmonary perfusion, has an influence on cardiopulmonary function, affecting both exercise capacity (measured as 6-MWD or VO_2_peak) and prognosis [15,16,17,18,19]. 

Similarly to the indices used for evaluation of COPD (chronic obstructive pulmonary disease), such as the BODE (body-mass, airflow obstruction, dyspnoea, and exercise capacity index), ADO (age, dyspnoea, and airflow obstruction index), and DOSE (dyspnoea, obstruction, smoking, and exacerbation index), various indices have been established for prognosis evaluation in IPF patients [20,21,22]. These indices consider gender, age, and lung function data to be prognostically relevant. However, these multidimensional indices did not outperform the single parameter ‘diffusion capacity’, which is evaluated by measuring the diffusing capacity for carbon monoxide (DLCO). Data for IPF patients have consistently shown that DLCO is the best individual prognostic marker, even outperforming forced vital capacity (FVC), and FVC and DLCO are known to represent the progression of high-resolution computed tomography (HRCT) findings in IPF patients [23].

The six-minute walk distance (6-MWD) evaluation is a simple exercise test with prognostic relevance in patients with ILD [24,25,26,27]. The 6-MWD and the associated oxygen desaturation show good correlation with VO_2_peak (mL/min/kg) and breathing efficacy (measured as the VÉ/VCO_2_ slope) in IPF patients [24]. This is also true for patients with sarcoidosis [28] and other ILDs [29,30,31]. In sarcoidosis patients, the peak oxygen uptake, maximum respiratory rate, breathing reserve, alveolar–arterial oxygen pressure gradient at peak exercise, and delta SpO_2_ values show a strong correlation with the relative differences in FVC %pred. and DLCO %pred. over five years [32]. 

Because of the frequent cardiovascular gas exchange defects and muscular comorbidities in ILD patients, cardiopulmonary exercise testing (CPET) has proven to be an elegant and relatively easy method to determine the individual performance and compensation ability of the respiratory system (lung–heart–circulation–muscle) and to differentiate the leading disturbances causing limitations of exercise capacity [33,34,35,36]. Against this background, CPET is used to evaluate intervention efficacy in pulmonary diseases [37]. The standardisation of CPET in ILD patients is a part of current investigations [38]. In the current literature, a couple of studies have demonstrated the prognostic relevance of CPET parameters in diverse ILD subgroups [39,40]. Nevertheless, a meta-analysis of 13 retrospective studies stated that there was insufficient evidence to confirm the value of CPET in facilitating ‘real-world’ clinical decision making in patients with ILD and that additional prospective studies are required to validate the putative prognostic associations reported in previous studies in carefully phenotyped patient populations [41].

Thus, the aim of this outpatient study was to assess the prognostic value of multiple CPET-derived parameters in a defined group of patients with ILD, with detailed analyses in IPF patients.

## 2. Material and Methods

### 2.1. Patients

In this single-centre retrospective study, a total of 215 patients with ILD were included. This centre follows the IPF-guidelines on diagnosis and therapy such as multidisciplinary discussion (MDD) [2,6,42]. No CPET data were available for 32 of the 215 patients. Therefore, the final study population consisted of 183 patients.

A definite/probable UIP (usual interstitial pneumonia) pattern was found in 55 of the 183 patients, and IPF was classified on the basis of this finding. Patients with CPFE syndrome were evaluated separately as a subgroup of patients with UIP [43,44]. In line with ATS/ERS guidelines [1], patients with a non-UIP pattern were classified as showing ‘sarcoidosis with fibrosis’ [45], ‘exogen-allergic alveolitis with fibrosis’ [46], or other conditions, including fibrotic non-specific interstitial pneumonia (NSIP) [47], fibrotic ‘interstitial pneumonia with autoimmune features’ (IPAF) [48], or unclassifiable ILD [49].

For the characterisation of the patients, sex, age, body mass index (BMI), and number of comorbidities (according to the Charlson comorbidity Index [50]) were analysed. The Charlson comorbidity Index predicts 10-year survival in patients focusing on 19 comorbidities with different assessment scores. The severity grading of IPF patients followed the modified GAP index [21].

### 2.2. Lung Function and Diffusing Capacity

Lung function parameters were calculated according to normative values, as described previously [51,52,53]. Obstructive pulmonary disease was defined as forced expiratory volume in 1 s (FEV1)/FVC < 70%; restrictive pulmonary disease by total lung capacity (TLC) < 80%; and clinically relevant diffusion impairment by DLCO < 60%.

### 2.3. Cardiopulmonary Exercise Testing

CPET was performed according to the modified JONES-protocol using a bicycle ergometer as a symptom-limited test. Performance and analysis methods have been previously described in detail [54]. Briefly, the test started with a 3-min resting phase and unloaded cycling of 1 min followed by a protocol with a step-increment protocol of 16 W∙min^−1^.

### 2.4. Right Heart Catheterisation

Right heart catheterisation (RHC) was performed in accordance with the guidelines of the ESC/ERS [55] and German recommendations [56]. PH was defined as mean pulmonary artery pressure (PAPmean) >20 mmHg, and pulmonary arterial hypertension was defined as PAPmean >20 mmHg, pulmonary artery wedge pressure (PAWP) ≤15 mmHg, and pulmonary vascular resistance (PVR) ≥3 Wood units (≥240 dyn∙s∙cm^−5^) [57].

### 2.5. Echocardiography

Resting echocardiography was performed by experienced physicians according to relevant guidelines [58,59]. TR was classified according to the American College of Cardiology/European Society of Cardiology (ESC) recommendations, and PAPsys was estimated using a simplified Bernoulli equation via TR velocity (v) as RVsys (mmHg) = 4v2, with the addition of 5 mmHg if the inferior vena cava was not dilated and there was visible respiratory variability and 10 mmHg if the inferior vena cava was dilated or without respiratory variability.

### 2.6. Follow-Up Assessments

The patients were contacted by phone and provided written informed consent for data collection. The date of evaluation was 01.03.2020 (mean observation time, 3.0 ± 2.5 years). The study was approved by the ethics committee of the University of Greifswald (Reg.-Nr. BB 057/2017). 

### 2.7. Methodological Limitations

The selection of patients was inherently biased, since only patients who underwent CPET were included in the analyses. As a result, despite the retrospective nature of the study, only five patients were lost to follow-up (2.7%). Moreover, due to the retrospective approach, not all clinical and functional data were available. Due to the fact that our institution is a supra-regional centre for PH-diagnosis and therapy, the proportion of PH-patients in this study is high. 

In all patients, the modified JONES-protocol was used on a braked cycle ergometer, which has been only evaluated for COPD patients so far and did not significantly influence the comparability of exercise parameters to other protocols [60,61]. Studies comparing different exercise protocols are not known for patients with ILD. Moreover, the current study focused on the prognostic evaluation of CPET parameters. Therefore, data on therapy are not provided. However, the effect of available antifibrotic medication on pulmonary function and cardiopulmonary exercise capacity is undisputable [8,62,63,64]. 

### 2.8. Statistical Analyses

Continuous variables, stratified by group status, were reported as the median and interquartile range (IQR, in brackets). Categorical variables were reported as absolute numbers and percentages. Differences among groups were verified using Wilcoxon (continuous data) and χ^2^ tests (categorical data). Potential associations of group status and parameters from pulmonary function testing and CPET with mortality were tested using Cox regression models adjusted for age and sex. For group status, the follow-up duration was calculated based on the time of diagnosis; for the other variables, the time of first examination was defined as the starting point. Prediction models were determined using Cox regression models with age, sex, body mass index (BMI), and all parameters from pulmonary function testing and CPET as explanatory variables. For the final model, variables using a backward selection procedure with a cut-off *p*-value of 0.1 were eliminated. The discrimination of these models was reported using Harrell’s C-statistic. Based on logistic regression models with the outcome ‘death: yes/no’, conducted receiver operating characteristic (ROC) analyses for selected variables were conducted. Kaplan–Meier curves were plotted for selected variables; for continuous variables, cut-off values were defined as the point which maximised the Youden index for the outcome ‘death’. The Youden index was defined as sensitivity + specificity − 1. All analyses were carried out using Stata 14.1 (Stata Corporation, College Station, TX, USA).

## 3. Results

### 3.1. Patient Characteristics 

The median age of the included patients (*n* = 183, 68% male) was 68.1 ± 10.4 years. The median age of patients with a UIP pattern was 72.8 ± 7.9 years and that of those with CPFE syndrome was 71.1 ± 8.3 years (Table 1). In the latter group, 95% of the patients were men. Patients with EAA (64.3 ± 10.5 years) and sarcoidosis (60.6 ± 12.1 years) were significantly younger than those with IPF (*p* < 0.001). The percentage of male patients was the smallest in the sarcoidosis group (53%). At the time of study inclusion, the diagnosis had been established for 2.7 ± 6.1 years in ILD patients and 2.0 ± 1.7 years in CPFE syndrome patients.

**Figure 1:**
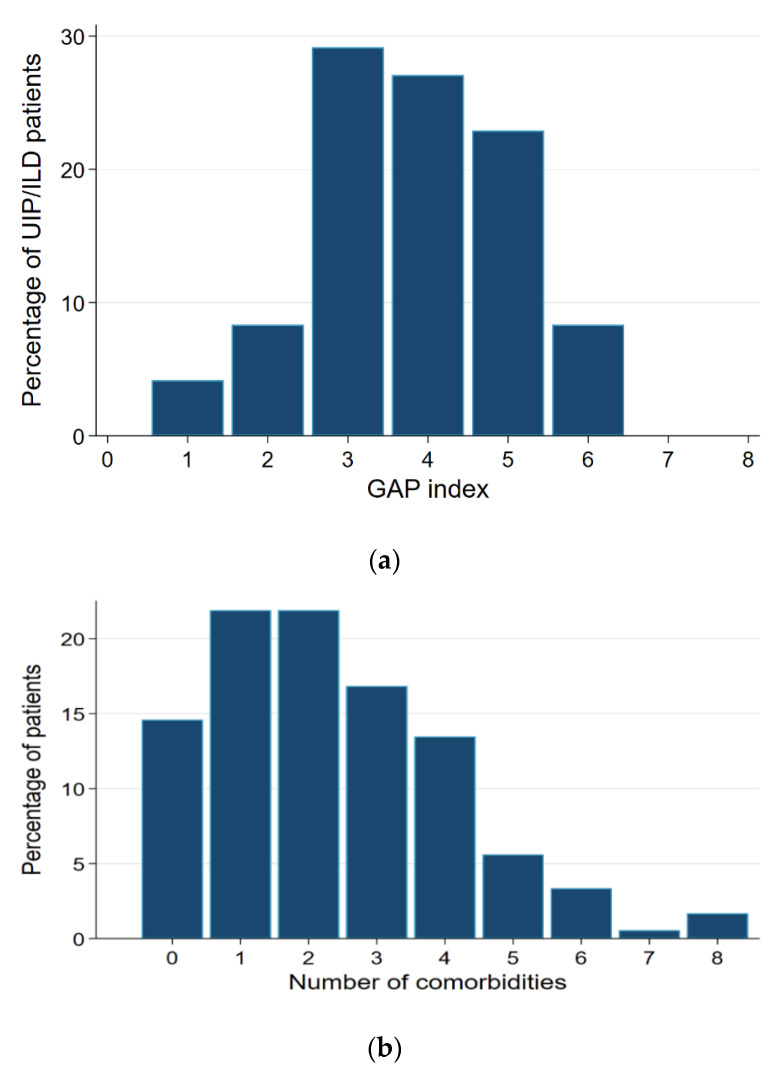
(**a**) The GAP-Index in IPF-patients (*n* = 48). UIP: Usual interstitial pneumonia; ILD: interstitial lung disease. (**b**) Number of comorbidities (in percent).

The average Charlson comorbidity index of the patients was 2 ± 2 points. The mean number of comorbidities was 2.4, and more than three comorbidities were reported in 41% of the patients (Figure 1). The GAP index in the IPF subgroup was ≥3 in 88% of the cases (Figure 1a).

### 3.2. Echocardiography 

On average, 71% of all patients had normal left ventricular function, and the corresponding value in patients with the UIP pattern was 59%. Reduced left ventricular function was documented in 11% of the patients. Right ventricular function was comparable in all patients, with an approximate tricuspid annular plane systolic excursion (TAPSE) of 21 ± 5 mm (Table 1).

### 3.3. Right Heart Catheterisation 

Haemodynamic data were available in a subgroup of 87 patients, and PH was diagnosed in 79% of these patients (68% of IPF patients and up to 100% of patients with sarcoidosis), Table 1. The RHC and non-RHC groups showed significant differences (Supplementary Table S1), especially in relation to the time between diagnosis and inclusion in the present study (1.3 ± 5.4 vs. 3.0 ± 6.8 years, *p* = 0.008). Patients who underwent invasive diagnostic procedures (RHC, *n* = 87) experienced diverse comorbidities more often (e.g., arterial hypertension, atrial fibrillation, renal insufficiency, coronary artery disease, venous thromboembolic disease). PH was known beforehand in 77% of patients who received RHC (Supplementary Table S1).

Although the RHC group and overall study group showed no significant differences in echocardiographic (except for PAPsyst: 33.4 ± 11.0 vs. 58.3 ± 19.9 mmHg for the RHC group, *p* < 0.001) and lung functional findings (except for DLCO and KCO% pred., *p* < 0.001), the RHC group showed poorer findings for CPET parameters such as performance (97 ± 32 vs. 68 ± 27 W, *p* < 0.001), oxygen uptake (17.1 ± 4.6 vs. 11.4 ± 3.3 mL/kg/min, *p* < 0.001), and breathing efficacy (VÉ/VCO_2_ slope: 37.9 ± 10.2 vs. 50.7 ± 15.5, *p* < 0.001).

### 3.4. Lung Function Values 

The overall group of patients had FEV_1_ (%pred.), FVC (%pred.), TLC value (%pred.), and DLCO value (%pred.) values of 81% ± 22%, 81% ± 22%, 79% ± 20%, and 44% ± 26%, respectively. The proportion of patients with TLC (%pred.) < 80%, FVC (%pred.) < 80%, DLCO (%pred.) < 60%, and KCO (%pred.) < 60% was 54%, 29%, 82%, and 49%, respectively (Table 1).

### 3.5. Cardiopulmonary Exercise Testing

The determined maximum power in watts (W) was 67% ± 30% pred., peak oxygen uptake was 62% ± 21% pred., and the VÉ/MVV ratio was 68% ± 21% pred. In 36 (20%) patients of the overall group, this value was >80% and, therefore, demonstrated pulmonary exercise limitation. The respiratory efficacy (measured as the VÉ/VCO_2_ slope) was 44 ± 14 in 77% of the patients, with values > 34 considered pathological. The PaetCO_2_ max value > 6 mmHg at the end of exercise demonstrated an inhomogeneity of ratio perfusion/ventilation and was pathological in 73% of the overall group. Interestingly, 31% of all ILD patients showed dynamic hyperinflation (defined as EELVmax − EELFrest > 0); in sarcoidosis patients, it was even evident in 65% of the patients (Table 1).

### 3.6. Survival

**Figure 2:**
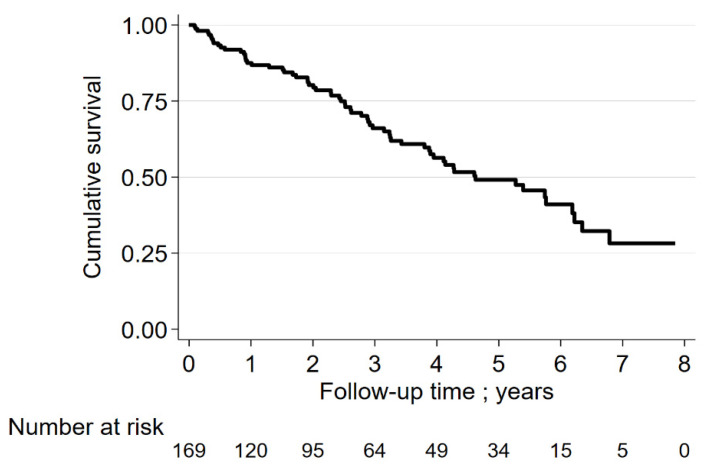
Survival rate of ILD-patients.

**Figure 3:**
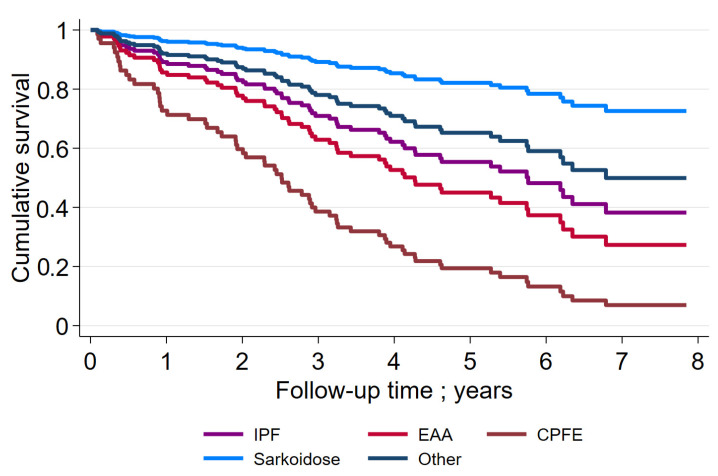
Survival rate in subgroups of ILD-patients. IPF: idiopathic pulmonary fibrosis; EAA: exogen allergic alveolitis; CPFE: combined pulmonary fibrosis and emphysema.

Figure 2 depicts the survival rates of the entire group of ILD patients. The 3-year and 5-year survival rates were 68% and 50%, respectively. Figure 3 shows the survival rates of subgroups of ILD patients. The 3-year and 5-year survival rates were the lowest in patients with CPFE. For IPF patients, the rates were 72% and 58%, respectively.

### 3.7. Parameters Relevant to Prognosis

Parameters relevant to prognosis over the years were determined by Cox regression analyses of the data at study entry (adjusted for age, sex, and body mass index, Supplementary Table S2). Three models were established:

**Model 1** (dyslipidaemia, PHT (medical history), TAPSE, PAPsys, FEV1 pred., and DLCO pred. (or KCO pred., *n* = 98). Using backward elimination, significance was observed for PAPsys (HR, 1.03; 95% CI, 1.01–1.04; *p* = 0.003) and TAPSE (HR, 0.88; 95% CI 0.82–0.96; *p* = 0.004). The C-statistic was 0.810.

Subgroup analyses in IPF patients (*n* = 55, mean age 72.8 ± 7.9 years, 76% male revealed a significant influence of dyslipidaemia (HR, 25.65; 95% CI 1.71–385.15; *p* = 0.019), PH (medical history) (HR, 31.73; 95% CI 3.15–319.91; *p* = 0.003), TAPSE (HR, 0.82; 95% CI 0.68–0.98; *p* = 0.032), and DLCO (%pred.) (HR, 1.02; 95% CI 1.00–1.04, *p* = 0.032). The C-statistic was 0.928.

**Model 2** (CPET values, without AaDO_2_ and PaetCO_2_, *n* = 134) demonstrated a significant influence of max. power (%pred.) (HR, 0.98; 95% CI 0.97–0.99; *p* = 0.039), petCO_2_ at rest (HR, 1.16; 95% CI 1.04–1.29; *p* = 0.006), VE/VCO_2_ @AT (HR, 1.09; 95% CI 1.03–1.16; *p* = 0.002), and VO_2_peak (%pred.) (HR, 0.97; 95% CI 0.94–0.99; *p* = 0.038) upon prognosis. The C-statistic was 0.826.

Subgroup analyses in IPF patients revealed prognostic significance for petCO_2_ at rest (HR, 1.45; 95% CI 1.14–1.86; *p* = 0.003), petCO_2_@AT (HR, 0.59; 95% CI 0.43–0.80; *p* = 0.001), and VO_2_peak (%pred.) (HR, 0.92; 95% CI 0.87–0.97; *p* = 0.001). The C-statistic was 0.869.

**Model 3** (comorbidities of medical history, patient characteristics (BMI, gender, and age), lung function, diffusing capacity, and CPET values without AaDO_2_ and PaetCO_2_, *n* = 112). This model demonstrated a significant influence of dyslipidaemia (HR, 3.37; 95% CI 1.19–9.57; *p* = 0.023), BMI (HR, 0.81; 95% CI 0.72–0.91; *p* = 0.000), RV (%pred.) (HR, 0.95; 95% CI 0.90–0.99; *p* = 0.023), TLC (%pred.) (HR, 1.09; 95% CI 1.00–1.20; *p* = 0.042), FEV1 (%pred.) (HR, 0.94; 95% CI 0.90–0.98; *p* = 0.008), VO_2_peak (mL/kg/min) (HR, 0.75; 95% CI 0.63–0.89; *p* = 0.001), petCO_2_@AT (mmHg) (HR, 0.74; 95% CI 0.63–0.87; *p* = 0.000), and petCO_2_ at rest (mmHg) (HR, 1.51; 95% CI 1.22–1.87; *p* = 0.000). The C-statistic was 0.869 (better than that of models 1 and 2).

**Figure 4:**
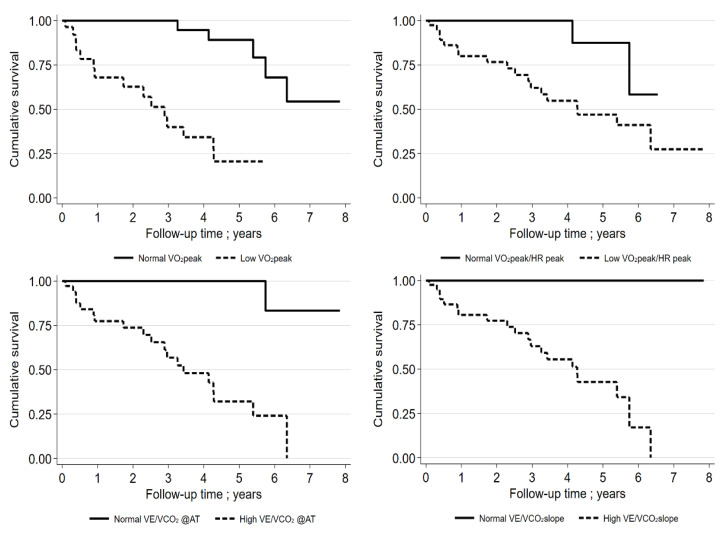
Kaplan–Meier curves for IPF patients (VO_2_peak % pred cut-off: 61%; VO_2_peak/HRpeak cut-off: 8.6 mL; VE/VCO_2_@AT cut-off: 39; VE/VCO_2_ slope cut-off: 40). “Low“ means beneath cut-off, “normal“ means above cut-off.

The IPF patient subgroup was too small for these analyses. In two of the three models, VO_2_peak (as mL/kg/min or %pred.) in the entire group of patients with ILD was of significant prognostic relevance. For the entire group, the best cut-off VO_2_peak was 61% pred. for the discrimination of mortality (for VÉ/VCO_2_@AT, 39; for VÉ/VCO_2_ slope, 40; and for VO_2_peak/HRpeak, 8.6 mL) (Supplementary Table S3, Figure 4).

In the subgroup of IPF patients (*n* = 55; mean age, 72.8 ± 7.9; 76% males), the best cut-off for VO_2_peak was 69% pred. for the discrimination of mortality (for VÉ/VCO_2_@AT, 37; for VÉ/VCO_2_ slope, 33; and for VO_2_peak/HRpeak, 13 mL). An additional analysis of the prognostic relevance of the GAP index (including the FEV values) was performed and was found to be significant for survival (HR, 2.44; 95% CI 1.5–3.96; *p* < 0.001). The C-statistic was 0.760. Only VO_2_peak %pred. remained statistically significant in the model with the GAP index, VO_2_peak %pred., and VÉ/VCO_2_ slope (HR, 0.93; 95% CI 0.88–0.98; *p* = 0.006). The C-statistic was found to be 0.883. Interestingly, FVC (%pred.) did not significantly influence the survival of patients with IPF (HR, 0.979; 95% CI 0.955–1.003; *p* = 0.087).

For the subgroup of patients who underwent RHC, the prognostic relevance of the presence of PH (defined as PAPmean > 20 mmHg) was examined (adjusted for age, sex, and BMI) but did not prove to be of significance for survival (HR, 2.3; 95% CI 0.90–5.94; *p* = 0.082).

## 4. Discussion

Our study with 183 ILD patients clearly demonstrated that the maximum oxygen uptake (VO_2_peak) had a highly significant prognostic influence. The best mortality predictive cut-off value for VO_2_peak in ILD patients was 61% pred., while in IPF patients, it was 69% pred. Only four studies included more than 100 patients. However, these studies lacked detailed information on comorbidities, and the included exercise variables were limited [41].

Dyslipidaemia, as well as the existence of PH (medical records), echocardiographically measured right ventricular function (TAPSE), and PAPsys were of prognostic significance. In addition to male sex and age > 70 years, the degree of dyspnoea; DLCO < 60% pred., 6-MWD < 250 m, and SpO_2_ < 88% measured during the 6-MWD; and the existence of PH and cardiovascular comorbidities were considered prognostically relevant baseline data in IPF patients [13,65,66]. Additionally, in the literature, histological data (number of fibroblastic foci), the extent of fibrotic alterations measured with HRCT, and selected biomarkers were prognostically relevant. Some of the prognostically relevant data are also reflected by our data, especially dyslipidaemia and PH. Against this background, it appears strange that comorbidities were not regularly part of investigations of ILD prognosis. With regard to dyslipidaemia, there were no differences between IPF and NSIP patients (20% and 17%) [7]. This is similar to our data. Although the prognostic significance of PH (medical history) is adequately familiar [15,67] it has not yet been described for dyslipidaemia in ILD-patients.

Focusing on lung function, only FEV1 and DLCO (as well as KCO) were reported to be prognostically relevant in our Cox analysis. In contrast to other studies [39,40,68], the prognostic significance for FVC was not observed. However, a good correlation between FVC and FEV1 is known [10]. The small number of included patients with IPF (*n* = 55/183) might explain the missing prognostic relevance of FVC.

The prognostic significance of diffusion parameters in ILD patients is undisputable [24,39], justifying their inclusion in prognosis scores [22]. DLCO is a sensitive marker for gas-exchange pathologies in fibrotic lung diseases. However, it is decisively influenced by the capillary blood volume [69]. Against this background, it is a good parameter for the detection of early pulmonary vascular injury [70]. It is reduced in manifest vascular disturbances and often associated with PH. In our study, 94% of the patients with PH showed pathological diffusion capacity (DLCO < 60% pred.), while the corresponding percentage in evaluations based on KCO pred. was 83%. The optimal prognostic cut-off value was 36% for DLCO pred. and 48% for KCO pred.

The prognostic relevance of CPET parameters for diverse subgroups of patients with ILD is commonly known [41]. In contrast to the straightforward and inexpensive 6-MWD, the most convincing advantage of CPET is the early detection of pathophysiological conditions and cardiopulmonary limitations. The limited lung compliance in ILD patients leads to only a small increase in breathing volume (Vt), which results in an increase in breathing frequency. Predominant dead-space ventilation leads to a pathological breathing efficacy. Hyperventilation is enhanced by the stimulation of mechanoreceptors of the lung. Additionally, the alveolo-arterial oxygen difference (AaDO_2_) at rest is often elevated in patients with ILD and increases during exercise due to oxygen exploitation in the muscle tissue. This eventually results in acidosis, which aggravates hyperventilation and impairs breathing efficacy. Impaired oxygen exchange in the lung eventually limits oxygen supply to the muscle tissue and exercise performance. The increased breathing effort claims a relevant proportion of oxygen uptake that is no longer available to the peripheral muscles. Depending on the extent of disease, it is comprehensible that ventilation, perfusion, and gas exchange are impaired in ILD patients [14,71]. During exercise, despite lung parenchymal disease, the heart rate is elevated and the stroke volume is reduced in ILD patients. Our data support these statements and prove the prognostic relevance of these parameters (Supplementary Tables S1 and S2).

Independent of the primary disease, which was also true for ILD patients, the existence of PH resulted in typical CPET changes: reduced VO_2_peak < 60% pred., increased VÉ/VCO_2_ slope > 45, PetCO_2_ < 33 mmHg at rest, and an increase of <3 mmHg during exercise [15,17,18,19,72,73]. Therefore, our PH patients showed a VO_2_peak of 47.4% ± 15.3% pred., a VÉ/VCO_2_ slope of 53.9 ± 15.9, and a PetCO_2_ at rest of 26.1 ± 5.5 mmHg. Interestingly, our analyses did not reveal a significant influence of invasively measured PH on survival in ILD patients. However, other ILD-studies (especially IPF studies) have described the prognostic relevance of PH in these patients [74,75,76,77].

In summary, the present data revealed that VO_2_peak pred., VÉ/VCO_2_ slope, and VÉ/VCO_2_@AT are of prognostic relevance in ILD patients (including those with IPF). This is consistent with other studies [41,78]. Surprisingly, in our patients, dyslipidaemia was prognostically relevant, but has not been described to show prognostic significance in ILD patients so far. None of the established prognostic parameters, including FVC %pred. and DLCO/KCO %pred., other than the VO_2_peak %pred. were shown to be relevant prognostic markers in the entire group of patients with ILD.

## Figures and Tables

**Table 1:** Data of patients with different interstitial lung diseases.

Parameter		Total		IPF		EAA		CPFE		Sarcoidosis		Other
		*n* = 183		*n* = 55		*n* = 30		*n* = 22		*n* = 19		*n* = 57
	*N*		*N*		*N*		*N*		*N*		*N*	
Age (years)		68.1 ± 10.4		72.8 ± 7.9		64.3 ± 10.5		71.1 ± 8.3		60.6 ± 12.1		66.8 ± 10.6
Female		59 (32%)		14 (24%)		11 (37%)		1 (5%)		9 (47%)		24 (42%)
Height (cm)		171 ± 10		170 ± 9		170 ± 9		174 ± 7		175 ± 13		170 ± 9
Weight (kg)		82 ± 17		80 ± 15		83 ± 16		82 ± 17		87 ± 29		81 ± 15
BMI (kg/m^2^)		28.0 ± 4.8		27.6 ± 3.8		28.5 ± 5.2		27.0 ± 4.9		28.1 ± 7.3		28.3 ± 4.5
GAP score	146	3.6 ± 1.5	48	3.8 ± 1.2	24	3.9 ± 1.5	17	4.4 ± 0.9	13	2.2 ± 1.5	44	3.4 ± 1.6
Time from diagnosis to CPET (years)	168	2.7 ± 6.1	50	1.5 ± 3.2	28	2.9 ± 5.3	20	0.0 ± 0.2	18	6.7 ± 14.0	52	1.5 ± 4.7
Time from CPET to Censoring (years)	180	3.0 ± 2.5	55	3.2 ± 2.4	30	3.3 ± 2.6	22	2.0 ± 1.7	19	2.7 ± 2.8	54	3.2 ± 2.7
Comorbidities												
Dyslipidemia		36 (20%)		16 (29%)		5 (17%)		3 (14%)		2 (11%)		10 (18%)
Diabetes mellitus		47 (26%)		17 (31%)		6 (20%)		4 (18%)		3 (16%)		17 (30%)
Arterial hypertension		104 (57%)		34 (62%)		17 (57%)		11 (50%)		8 (42%)		34 (60%)
Atrial fibrillation		34 (19%)		13 (24%)		5 (17%)		6 (27%)		1 (5%)		9 (16%)
Chronic heart failure		32 (17%)		11 (20%)		5 (17%)		3 (14%)		2 (11%)		11 (19%)
PAOD		8 (4%)		4 (7%)		1 (3%)		1 (5%)		0 (0%)		2 (4%)
Renal insufficiency		35 (19%)		14 (25%)		2 (7%)		5 (23%)		3 (16%)		11 (19%)
Pulmonary hypertension		69 (38%)		12 (22%)		15 (50%)		14 (64%)		7 (37%)		21 (37%)
Cancer		24 (13%)		10 (18%)		2 (7%)		3 (14%)		2 (11%)		7 (12%)
Coronary artery disease		47 (26%)		16 (29%)		5 (17%)		12 (55%)		2 (11%)		12 (21%)
COPD/Asthma		30 (16%)		5 (9%)		3 (10%)		8 (36%)		5 (26%)		9 (16%)
Venous thromboembolic disease		18 (10%)		5 (9%)		2 (7%)		1 (5%)		4 (21%)		6 (11%)
Cerebrovascular disease		13 (7%)		3 (5%)		2 (7%)		3 (14%)		0 (0%)		5 (9%)
Charlson Index	183	2.1 ± 2.0	55	2.4 ± 2.1	30	1.4 ± 1.5	22	2.6 ± 2.8	19	1.4 ± 1.4	57	2.1 ± 2.0
Echocardiography												
LVEF	151		41		25		19		15		50	
normal, >55%		107 (71%)		24 (59%)		18 (72%)		15 (79%)		13 (81%)		37 (74%)
reduced, <45%		16 (11%)		7 (17%)		1 (4%)		3 (16%)		1 (6%)		4 (8%)
TAPSE (mm)	148	21 ± 5	44	20 ± 5	26	22 ± 5	18	20 ± 5	17	22 ± 4	43	22 ± 5
TI	169	124 (73%)	48	37 (77%)	28	20 (71%)	20	18 (90%)	17	12 (71%)	56	37 (66%)
Estimated PAPsys (mmHg)	124	47 ± 21	37	42 ± 16	20	54 ± 25	18	57 ± 18	12	38 ± 25	37	46 ± 19
Right heart catheter			19		16		16					
RAPmean (mmHg)	84	6.4 ± 4.4	18	5.2 ± 4.2	16	7.3 ± 2.8	16	6.4 ± 5.0	5	6.0 ± 3.3	29	6.8 ± 5.1
PAPmean (mmHg)	86	35.3 ± 13.0	19	28.6 ± 11.0	16	37.9 ± 14.5	16	38.1 ± 10.9	5	37.4 ± 6.8	30	36.3 ± 14.3
PAPmean > 20 mmHg and PVR ≥ 3	84	66 (79%)	19	13 (68%)	16	15 (94%)	16	13 (81%)	4	4 (100%)	29	21 (72%)
PAWP (mmHg)	86	9.9 ± 5.7	19	7.9 ± 4.2	16	9.9 ± 4.6	16	11.6 ± 7.4	4	7.8 ± 3.9	30	10.4 ± 6.0
PVR (WU)	84	6.8 ± 5.2	19	5.5 ± 3.7	16	7.5 ± 6.5	16	6.3 ± 4.0	4	6.7 ± 2.3	29	7.5 ± 6.1
CO/Thermo (L)	87	4.4 ± 1.5	19	4.4 ± 1.3	16	4.5 ± 1.2	17	4.9 ± 1.7	5	4.3 ± 1.1	30	4.2 ± 1.6
CI (L/min/m^2^)	87	2.3 ± 0.7	19	2.4 ± 0.6	16	2.4 ± 0.8	17	2.4 ± 0.7	5	2.3 ± 0.5	30	2.2 ± 0.7
Lung function testing												
TLC (%pred.)	179	79 ± 20	54	76 ± 22	29	74 ± 17	21	90 ± 11	19	87 ± 19	56	79 ± 20
reduced, <80%	179	96 (54%)	54	35 (65%)	29	20 (69%)	21	5 (24%)	19	6 (32%)	56	30 (54%)
VC (%pred.)	178	77 ± 22	53	80 ± 23	29	69 ± 23	21	90 ± 13	19	77 ± 17	56	74 ± 22
reduced, <80%	178	94 (53%)	53	25 (47%)	29	20 (69%)	21	5 (24%)	19	12 (63%)	56	32 (57%)
FVC (%pred.)	179	81 ± 22	54	84 ± 23	29	71 ± 21	21	94 ± 14	19	85 ± 18	56	76 ± 23
reduced, <80%	179	52 (29%)	54	13 (24%)	29	12 (41%)	21	0 (0%)	19	4 (21%)	56	23 (41%)
FEV1 (%pred.)	179	81 ± 22	54	90 ± 21	29	72 ± 18	21	88 ± 17	19	78 ± 22	56	76 ± 24
FEV1/FVC (%)	180	80 ± 13	54	83 ± 9	29	80 ± 18	21	71 ± 10	19	74 ± 12	56	82 ± 12
RV (%pred.)	179	87 ± 35	54	73 ± 37	29	85 ± 25	21	93 ± 27	19	107 ± 32	57	93 ± 36
RV/TLC (%pred.)	178	42 ± 13	53	37 ± 11	29	43 ± 8	21	39 ± 8	19	43 ± 7	56	46 ± 17
DLCO (%pred.)	146	44 ± 26	48	47 ± 35	24	38 ± 16	17	30 ± 11	13	58 ± 20	44	46 ± 20
reduced, <60% pp	146	120 (82%)	48	42 (88%)	24	21 (88%)	17	16 (94%)	13	6 (46%)	44	35 (80%)
KCO (%pred.)	151	61 ± 22	48	63 ± 17	24	57 ± 18	18	41 ± 13	15	72 ± 30	46	64 ± 25
reduced, <60%	151	74 (49%)	48	21 (44%)	24	13 (54%)	18	16 (89%)	15	4 (27%)	46	20 (43%)
CPET												
Max. performance Watt	183	83 ± 33	55	83 ± 29	30	77 ± 29	22	76 ± 31	19	106 ± 45	57	82 ± 32
Max. performance (%pred.)	183	67 ± 30	55	67 ± 27	30	63 ± 31	22	49 ± 19	19	79 ± 27	57	71 ± 35
VO_2_peak (mL/min/kg)	183	14 ± 5	55	15 ± 5	30	14 ± 4	22	12 ± 4	19	17 ± 5	57	15 ± 5
VO_2_peak (%pred.)	183	62 ± 21	55	65 ± 20	30	58 ± 19	22	48 ± 15	19	69 ± 19	57	65 ± 23
VO_2_ @ AT (% share VO_2_peak pred.)	173	40 ± 11	54	41 ± 11	28	40 ± 12	21	33 ± 8	19	43 ± 9	57	42 ± 13
pathological, <40%	173	89 (51%)	54	24 (44%)	28	17 (61%)	21	17 (81%)	19	7 (37%)	57	24 (47%)
VO_2_/HR max. (mL/beat)	183	10.3 ± 4.7	55	11.5 ± 6.6	30	9.5 ± 3.2	22	8.4 ± 2.7	19	10.8 ± 4.2	57	10.0 ± 3.6
VÉ/VCO_2_ slope	168	44 ± 14	53	44 ± 14	26	45 ± 15	20	56 ± 16	19	35 ± 8	50	42 ± 13
pathological, >34	168	130 (77%)	53	43 (81%)	26	20 (77%)	20	19 (95%)	19	13 (68%)	50	35 (70%)
VÉ/VCO_2_ rest	179	48 ± 11	54	47 ± 9	29	48 ± 11	20	56 ± 11	19	44 ± 7	56	47 ± 11
VÉ/VCO_2_ @ AT	168	44 ± 12	53	44 ± 11	26	42 ± 10	20	54 ± 13	19	38 ± 7	50	43 ± 13
petCO_2_ rest (mmHg)	174	28 ± 5	53	29 ± 4	29	28 ± 5	19	24 ± 4	19	29 ± 4	54	29 ± 5
petCO_2_ @ AT (mmHg)	169	30 ± 6	52	30 ± 5	27	30 ± 7	19	24 ± 6	19	33 ± 5	54	30 ± 6
AaDO_2_ max (mmHg)	139	57 ± 17	44	55 ± 15	23	66 ± 14	18	68 ± 16	15	41 ± 15	39	55 ± 15
pathological, >35	139	125 (90%)	44	41 (93%)	23	23 (100%)	18	16 (89%)	15	9 (60%)	39	36 (92%)
PaetCO_2_ rest (mmHg)	148	8.3 ± 4.9	45	8.3 ± 4.8	23	7.9 ± 5.0	19	11.0 ± 4.4	19	6.9 ± 3.5	42	7.9 ± 5.4
PaetCO_2_ max (mmHg)	133	9.7 ± 5.5	42	9.7 ± 4.5	21	11.4 ± 6.0	18	13.3 ± 4.3	15	3.5 ± 4.1	37	9.4 ± 5.5
pathological, >6	133	97 (73%)	42	34 (81%)	21	17 (81%)	18	17 (94%)	15	3 (20%)	37	26 (70%)
VÉ/MVV (%)	179	68 ± 21	54	63 ± 20	28	71 ± 19	21	64 ± 17	19	64 ± 18	57	73 ± 24
pathological, >80%	179	36 (20%)	54	6 (11%)	28	8 (28%)	21	2 (10%)	19	2 (11%)	57	18 (32%)
IC max − IC rest (L)	155	0.02 ± 0.49	48	0.19 ± 0.50	25	0.00 ± 0.53	19	−0.18 ± 0.48	18	−0.04 ± 0.45	45	−0.04 ± 0.42
pathological, <0	155	81 (52%)	48	16 (33%)	25	16 (64%)	19	12 (63%)	18	11 (61%)	45	26 (58%)
EELV max − EELV rest (L)	153	−0.13 ± 0.35	48	−0.20 ± 0.31	25	−0.09 ± 0.33	19	−0.23 ± 0.42	17	0.10 ± 0.40	44	−0.13 ± 0.31
pathological, >0	153	48 (31%)	48	13 (27%)	25	9 (36%)	19	5 (26%)	17	11 (65%)	44	10 (23%)
BF rest (/min)	183	22.3 ± 8.0	55	21.1 ± 7.3	30	24.0 ± 9.1	22	21.3 ± 6.2	19	18.3 ± 5.7	57	24.2 ± 8.7
BF max (/min)	183	37.7 ± 10.3	55	37.1 ± 11.1	30	39.4 ± 10.6	22	34.0 ± 8.6	19	33.0 ± 6.1	57	40.5 ± 10.3
VT rest (L)	183	0.77 ± 0.34	55	0.74 ± 0.31	30	0.80 ± 0.40	22	0.85 ± 0.39	19	0.91 ± 0.47	57	0.70 ± 0.24
VT max (L)	183	1.6 ± 0.6	55	1.6 ± 0.5	30	1.6 ± 0.7	22	2.0 ± 0.6	19	1.8 ± 0.7	57	1.50 ± 0.60
VÉ max (L/min)	183	58.1 ± 16.9	55	57.9 ± 15.8	30	57.0 ± 18.1	22	63.5 ± 16.6	19	57.7 ± 19.1	57	57.0 ± 16.8
HR rest (bpm)	183	80 ± 16	55	75 ± 19	30	81 ± 15	22	78 ± 16	19	85 ± 11	57	83 ± 15
HR max (bpm)	183	117 ± 22	55	110 ± 25	30	120 ± 19	22	113 ± 20	19	133 ± 17	57	120 ± 21
SysBP rest (mmHg)	172	115 ± 17	49	114 ± 16	30	114 ± 17	22	117 ± 17	17	116 ± 16	54	115 ± 18
SysBP max (mmHg)	180	138 ± 34	53	127 ± 23	30	138 ± 41	22	134 ± 28	19	167 ± 39	56	140 ± 34
DiasBP rest (mmHg)	175	74 ± 13	50	71 ± 13	30	76 ± 14	22	73 ± 13	17	79 ± 12	56	75 ± 11
DiasBP max (mmHg)	181	77 ± 18	53	72 ± 13	30	76 ± 15	22	74 ± 14	19	95 ± 14	57	78 ± 22

BMI: body mass index (kg/m^2^); CPET: cardiopulmonary exercise testing; IPF: idiopathic pulmonary fibrosis; EAA: exogen allergic alveolitis; CPFE: combined pulmonary fibrosis and emphysema; PAOD: peripheral arterial occlusive disease; COPD: chronic obstructive pulmonary disease; LVEF: left vetricuar ejection fraction (%); TAPSE: tricuspid annular plane systolic excursion (mm), TI: tricuspid insufficiency; PAP: pulmonary artery pressure (mmHg); PAWP: pulmonary artery wedge pressure (mmHg); PVR: pulmonal vascular resistance (WU); CO: cardiac output (L); CI: cardiac index (L/min/m^2^); TLC: total lung capacity (L); VC: vital capacity (L); FVC: forced vital capacity (L); FEV1: forced expiratory volume in 1 s (L); RV: residual volume (L); DLCO: diffusion capacity (mmol/min/kPa); KCO: global diffusion capacity (mmol/min/kPa/L); VO_2_: oxygen uptake (mL); AT: anaerobic threshold; HR: heart rate (bpm); VO_2_/HR: oxygen pulse (mL/beat); VÉ/VCO_2_: breathing efficacy; petCO_2_: end tidal carbon dioxide (mmHg); AaDO_2_: alveolar arterial oxygen difference (mmHg); PaetCO_2_: gradient between petCO_2_ and arterial CO_2_ levels (mmHg); VÉ/MVV: minute ventilation/maximum voluntary ventilation (%); IC: inspiratory capacity (L); EELV: endexpiratory lung volume (L); BF: breathing frequency (/min); VÉ: minute ventilation (L/min); VT: breathing volume (L); SysBP: systolic blood pressure (mmHg); DiasBP: diastolic blood pressure (mmHg).

## Data Availability

On request data can be made available.

## Supplementary Materials

The following supporting information can be downloaded at: https://www.mdpi.com/article/10.3390/jcm11061609/s1, Table S1: Patients with/without Right heart catheter; Table S2: Associations of measurements from medical history, echocardiography, right heart catheter, lung function testing and cardiopulmonary exercise testing with mortality; Table S3: Sensitivity, specificity, and Youden index for the perfect cut-offs for mortality (selected data).

## Abbreviations

ILD	interstitial lung disease
RHC	right heart catheter
CRF	cardiorespiratory fitness
BMI	body mass index
FVC	forced vital capacity
CPET	cardiopulmonary exercise testing
PH	pulmonary hypertension
COPD	chronic obstructive pulmonary disease
BODE	body-mass, airflow obstruction, dyspnea, and exercise capacity index
ADO	age, dyspnoea, airflow obstruction index
DOSE	dyspnoea, obstruction, smoking, exacerbation index
HRCT	high-resolution computed tomography
NSIP	non-specific interstitial pneumonia
UIP	usual interstitial pneumonia
IPAF	interstitial pneumonia with autoimmune features
DLCO	diffusion capacity
CPFE	combined pulmonary fibrosis and emphysema
VO_2_peak	peak oxygen uptake
VO_2_@AT	oxygen uptake at anaerobic threshold
VÉ/VCO_2_ slope	breathing efficacy
VÉ/VCO_2_@AT	breathing efficacy at anaerobic threshold
6-MWD	6-min walking distance
FEV_1_	forced expiratory volume in 1 s
SD	standard deviation
ROC	receiver operating characteristic
TLC	total lung capacity
W	Watt
RR	relative risk
CPI	composite physiological index
