# Supplementary material for: Value of Cardiopulmonary Exercise Testing in Prognostic Assessment of Patients with Interstitial Lung Diseases
Source: J Clin Med. 2022 Mar 14;11(6):1609. doi: 10.3390/jcm11061609 (PMC8954900; doi:10.3390/jcm11061609)
Supplement: Supplementary file 1 [file jcm-11-01609-s001.zip › Supplement table 1_JCM.pdf]

Table S1. Patients with/without Right heart catheter.

Parameter	without RHC	with RHC	PAPmean > 20 mmHg & PVR ≥ 3
	n=96	n=87	n=67
Age	68.1 ± 10.1	68.0 ± 10.9	67.7 ± 11.7
Female	29 (30%)	30 (34%)	23 (35%)
Height	172 ± 9	170 ± 10	169 ± 10
Weight	82 ± 17	82 ± 18	82 ± 18
BMI (kg/m²)	27.7 ± 4.7	28.2 ± 4.9	28.4 ± 4.9
Gap Score	3.2 ± 1.5	4.2 ± 1.3	4.2 ± 1.2
Time from diagnosis to CPET (years)	1.3 ± 5.4	3.0 ± 6.8	2.8 ± 5.5
Time from CPET to Censoring (years)	3.4 ± 2.5	2.6 ± 2.5	2.6 ± 2.6
**Comorbidities**			
Dyslipidemia	19 (20%)	17 (20%)	13 (20%)
Diabetes mellitus	23 (24%)	24 (28%)	21 (32%)
Arterial hypertension	48 (50%)	56 (64%)	42 (64%)
Atrial fibrillation	13 (14%)	21 (24%)	16 (24%)
Chronic heart failure	16 (17%)	16 (18%)	13 (20%)
PAOD	3 (3%)	5 (6%)	5 (8%)
Renal insufficiency	11 (11%)	24 (28%)	16 (24%)
Pulmonary hypertension	9 (9%)	60 (69%)	51 (77%)
Cancer	19 (20%)	5 (6%)	3 (5%)
Coronary artery disease	19 (20%)	28 (32%)	22 (33%)
COPD/Asthma	14 (15%)	16 (18%)	13 (20%)
Venous thromboembolic disease	7 (7%)	11 (13%)	8 (12%)
Cerebrovascular disease	6 (6%)	7 (8%)	5 (8%)
Charlson Index	1.8 ± 2.0	2.3 ± 2.1	2.2 ± 2.2
**Echocardiography**			
LVEF (%)	1.64 ± 0.63	1.56 ± 0.72	1.56 ± 0.71
normal, > 55 %	55 (72%)	52 (69%)	39 (68%)
reduced, < 45 %	6 (8%)	10 (13%)	7 (12%)
TAPSE (mm)	22.6 ± 4.7	19.8 ± 5.4	18.8 ± 5.5
TI	56 (66%)	68 (81%)	53 (82%)
Estimated PAPsys (mmHg)	33.4 ± 11.0	58.3 ± 19.9	61.3 ± 18.7
**Lung function testing**			
TLC (%pred)	79.1 ± 19.0	79.5 ± 20.7	78.6 ± 17.2
reduced, < 80 %	51 (55%)	45 (52%)	36 (55%)
VC (% pred.)	78.7 ± 23.8	75.5 ± 19.9	75.1 ± 18.2
reduced, < 80 %	47 (51%)	47 (55%)	37 (57%)
FVC (% pred.)	83.3 ± 22.4	77.9 ± 20.9	77.6 ± 19.0
reduced, < 80 %	22 (24%)	30 (35%)	21 (32%)
FEV1 (% pred.)	85.3 ± 22.5	77.0 ± 20.9	76.2 ± 19.5
FEV1/FVC (%)	80.8 ± 13.6	79.5 ± 12.2	78.4 ± 11.9
RV (% pred.)	84.1 ± 30.4	90.8 ± 38.8	89.6 ± 35.5
RV/TLC (% pred.)	39.8 ± 9.6	44.4 ± 14.9	44.5 ± 15.9
DLCO (% pred.)	49.8 ± 17.2	37.6 ± 31.9	35.7 ± 34.8
reduced, <60 % pp	61 (76%)	59 (89%)	49 (94%)
KCO (% pred.)	71.0 ± 18.6	49.4 ± 21.1	45.9 ± 17.5
reduced, < 60 %	21 (26%)	53 (75%)	44 (81%)
**Cardiopulmonary exercise testing**			
Max. performance Watt	97 ± 32	68 ± 27	64 ± 23
Max. performance (% pred.)	76 ± 32	56 ± 25	55 ± 26
VO_2 peak (ml/min/kg)	17.1 ± 4.6	11.4 ± 3.3	10.8 ± 2.9
VO_2 peak (% pred.)	73.0 ± 18.9	50.7 ± 16.2	47.8 ± 14.5
VO_2 @ AT (% share on VO_2 peak pred.)	44.3 ± 11.0	35.7 ± 9.8	34.2 ± 9.3

pathological, < 40 %	33 (35%)	56 (70%)	46 (77%)
VO_2/HR max. (ml/beat)	11.8 ± 5.4	8.5 ± 3.1	8.1 ± 2.6
VÉ/VCO_2 slope	37.9 ± 10.2	50.7 ± 15.5	53.8 ± 15.9
pathological, > 34	53 (55%)	64 (74%)	50 (76%)
VÉ/VCO_2 rest	45.5 ± 9.0	50.8 ± 11.5	53.3 ± 11.5
VÉ/VCO_2 @ AT	39.6 ± 9.3	48.7 ± 12.7	51.7 ± 12.8
petCO_2 rest (mmHg)	29.7 ± 4.0	27.0 ± 5.5	25.6 ± 4.9
petCO_2 @ AT (mmHg)	32.3 ± 5.2	26.8 ± 6.1	25.3 ± 5.6
AaD02 max (mmHg)	49.7 ± 14.9	65.5 ± 14.9	67.9 ± 12.8
pathological, > 35	65 (87%)	60 (94%)	47 (98%)
PaetCO_2 rest (mmHg)	6.6 ± 3.9	10.3 ± 5.2	10.8 ± 5.2
PaetCO_2 max (mmHg)	7.5 ± 5.0	12.2 ± 5.1	13.0 ± 5.1
pathological, > 6	43 (60%)	54 (89%)	42 (93%)
VÉ/MVV (%)	65.3 ± 20.9	70.3 ± 20.9	71.8 ± 19.9
pathological, > 80 %	17 (18%)	19 (22%)	15 (23%)
IC max - IC rest (l)	0.04 ± 0.48	0.00 ± 0.50	0.05 ± 0.49
pathological, < 0	41 (48%)	40 (58%)	28 (55%)
EELV max - EELF rest (l)	-0.14 ± 0.36	-0.12 ± 0.33	-0.10 ± 0.34
pathological, > 0	26 (30%)	22 (33%)	16 (33%)
BF rest (/min)	20.5 ± 7.1	24.2 ± 8.5	25.6 ± 8.7
BF max (/min)	36.4 ± 10.2	39.2 ± 10.2	39.2 ± 10.1
VT rest (l)	0.79 ± 0.38	0.74 ± 0.28	0.74 ± 0.28
VT max (l)	1.72 ± 0.62	1.52 ± 0.58	1.54 ± 0.55
VÉ max (l/min)	59.7 ± 16.3	56.4 ± 17.4	57.1 ± 16.6
HR rest (bpm)	80 ± 18	79 ± 14	78 ± 13
HR max (bpm)	123 ± 24	111 ± 19	110 ± 20
SysBP rest (mmHg)	116 ± 17	112 ± 17	112 ± 17
SysBP max (mmHg)	149 ± 34	126 ± 30	124 ± 29
DiasBP rest (mmHg)	75 ± 12	73 ± 14	73 ± 15
DiasBP max (mmHg)	78 ± 17	76 ± 19	75 ± 20

BMI: body mass index (kg/m²); CPET: cardiopulmonary exercise testing; PAOD: peripheral arterial occlusive disease; COPD: chronic obstructive pulmonary disease; LVEF: left vetricuar ejection fraction (%); TAPSE: tricuspid annular plane systolic excursion (mm), TI: tricuspid insufficiency; PAP: pulmonary artery pressure (mmHg); TLC: total lung capacity (l); VC: vital capacity (l); FVC: forced vital capacity (l); FEV1: forced expiratory volume in 1 second (l); RV: residual volume (l); DLCO: diffusion capacity (mmol/min/kPa); KCO: global diffusion capacity (mmol/min/kPa/l); VO_2: oxygen uptake (ml); AT: anaerobic threshold; HR: heart rate (bpm); VO_2/HR: oxygen pulse (ml/beat); VÉ/VCO_2: breathing efficacy; petCO_2: end tidal carbon dioxide (mmHg); AaDO_2: alveolar arterial oxygen difference (mmHg); PaetCO_2: gradient between petCO_2 and arterial CO_2 levels (mmHg); VÉ/MVV: minute ventilation/maximum voluntary ventilation (%); IC: inspiratory capacity (l); EELV: endexpiratory lung volume (l); BF: breathing frequency (/min); VÉ: minute ventilation (l/min); VT: breathing volume (l); sysBP: systolic blood pressure (mmHg); diasBP: diastolic blood pressure (mmHg).
