# Supplementary material for: Value of Cardiopulmonary Exercise Testing in Prognostic Assessment of Patients with Interstitial Lung Diseases
Source: J Clin Med. 2022 Mar 14;11(6):1609. doi: 10.3390/jcm11061609 (PMC8954900; doi:10.3390/jcm11061609)
Supplement: Supplementary file 1 [file jcm-11-01609-s001.zip › Supplement table 2_JCM.pdf]

**Table S2.** Associations of measurements from medical history, echocardiography, right heart catheter, lung function testing and cardiopulmonary exercise testing with mortality.

| Parameter | N | Hazard Ratio (95% Confidence Interval) |
|---|---|---|
| **Medical history** | | |
| Dyslipidemia | 178 | 1.78 (1.04; 3.04)* |
| Diabetes mellitus | 178 | 0.70 (0.40; 1.23) |
| Arterial hypertension | 178 | 1.34 (0.80; 2.26) |
| Atrial fibrillation | 178 | 1.30 (0.71; 2.36) |
| Chronic heart failure | 178 | 1.68 (0.90; 3.14) |
| PAOD | 178 | 1.67 (0.60; 4.62) |
| Renal insufficiency | 178 | 1.49 (0.84; 2.66) |
| Pulmonary hypertension | 178 | 3.78 (2.29; 6.20)* |
| Cancer | 178 | 0.64 (0.28; 1.43) |
| Coronary artery disease | 178 | 1.28 (0.74; 2.22) |
| COPD/Asthma | 178 | 1.21 (0.66; 2.24) |
| Venous thromboembolic disease | 178 | 1.13 (0.50; 2.53) |
| Cerebrovascular disease | 178 | 0.68 (0.24; 1.89) |
| Charlson index | 178 | 1.02 (0.91; 1.14) |
| GAP-score | 142 | 1.70 (1.26; 2.30) |
| **Echocardiography** | | |
| LVEF (%, reduced vs. normal) | 146 | 1.64 (0.89; 3.01) |
| TAPSE (mm) | 144 | 0.88 (0.83; 0.93)* |
| TI | 164 | 1.71 (0.90; 3.26) |
| Estimated PAPsys (mmHg) | 124 | 1.05 (1.03; 1.07)* |
| **Right heart catheter** | | |
| RAP mean (mmHg) | 82 | 1.05 (0.99; 1.12) |
| PAPmean (mmHg) | 84 | 1.02 (0.99; 1.04) |
| PAPmean > 20 mmHg & PVR ≥ 3 | 84 | 1.57 (0.70; 3.55) |
| PAWP (mmHg) | 84 | 1.01 (0.95; 1.06) |
| PVR (WU) | 82 | 1.05 (0.99; 1.11) |
| CO/Thermo (l) | 85 | 0.86 (0.67; 1.12) |
| CI (l/min/m²) | 85 | 0.81 (0.49; 1.34) |
| **Lung function testing** | | |
| TLC (%pred) | 174 | 0.991 (0.977; 1.004) |
| reduced, < 80 % | 174 | 1.06 (0.64; 1.76) |
| VC (% pred.) | 173 | 0.991 (0.978; 1.003) |
| reduced, < 80 % | 173 | 1.15 (0.70; 1.91) |
| FVC (% pred.) | 174 | 0.990 (0.978; 1.003) |
| FEV1 (% pred.) | 174 | 0.985 (0.973; 0.998)* |
| FEV1/FVC (%) | 174 | 0.989 (0.970; 1.008) |
| RV (% pred.) | 174 | 0.996 (0.987; 1.004) |
| RV/TLC (% pred.) | 173 | 0.998 (0.978; 1.018) |
| DLCO (% pred.) | 142 | 0.978 (0.960; 0.997)* |
| Patients with value </= 60 % pp | 142 | 1.65 (0.69; 3.94) |
| KCO (% pred.) | 147 | 0.962 (0.947; 0.977)* |
| **Cardiopulmonary exercise testing** | | |
| Max. Performance Watt | 178 | 0.969 (0.958; 0.980)* |
| Max. Performance (% pred.) | 178 | 0.966 (0.954; 0.978)* |
| $VO_2$ peak (ml/min/Kg) | 178 | 0.78 (0.72; 0.84)* |
| $VO_2$ peak (% pred.) | 178 | 0.942 (0.925; 0.958)* |
| $VO_2$ @ AT (% share on $VO_2$ peak pred.) | 168 | 0.960 (0.940; 0.980)* |

| | | |
|---|---|---|
| pathological, < 40 % | 168 | 3.05 (1.73; 5.37)* |
| $VO_2$/HR max. (ml/beat) | 178 | 0.76 (0.69; 0.84)* |
| VÉ/$VCO_2$ slope | 178 | 1.043 (1.028; 1.059)* |
| pathological, > 34 | 178 | 1.77 (1.02; 3.06)* |
| VÉ/$VCO_2$ rest | 174 | 1.056 (1.030; 1.081)* |
| VÉ/$VCO_2$ @ AT | 163 | 1.064 (1.042; 1.087)* |
| pet$CO_2$ rest (mmHg) | 169 | 0.939 (0.894; 0.987)* |
| pet$CO_2$ @ AT (mmHg) | 162 | 0.920 (0.883; 0.958)* |
| Aa$DO_2$ max (mmHg) | 135 | 1.048 (1.028; 1.069)* |
| pathological, > 35 | 135 | 4.02 (0.92; 17.66) |
| Paet$CO_2$ rest (mmHg) | 144 | 1.096 (1.046; 1.149)* |
| Paet$CO_2$ max (mmHg) | 129 | 1.096 (1.050; 1.146)* |
| pathological, > 6 | 129 | 2.81 (1.14; 6.89)* |
| VÉ/MVV (%) | 174 | 1.004 (0.992; 1.017) |
| pathological, > 80 % | 174 | 0.82 (0.42; 1.59) |
| IC max - IC rest (l) | 150 | 1.14 (0.65; 2.00) |
| pathological, < 0 | 150 | 1.10 (0.63; 1.90) |
| EELV max - EELF rest (l) | 148 | 1.44 (0.68; 3.07) |
| pathological, > 0 | 148 | 1.37 (0.77; 2.44) |
| BF rest (/min) | 178 | 1.06 (1.03; 1.10)* |
| BF max (/min) | 178 | 1.01 (0.98; 1.03) |
| VT rest (l) | 178 | 0.82 (0.34; 1.95) |
| VT max (l) | 178 | 0.62 (0.36; 1.06) |
| VÉ max (l/min) | 178 | 0.979 (0.962; 0.996) |
| HR rest (bpm) | 178 | 0.999 (0.984; 1.013) |
| HR max (bpm) | 178 | 0.982 (0.971; 0.994)* |
| SysBP rest (mmHg) | 168 | 1.014 (0.998; 1.030) |
| SysBP max (mmHg) | 175 | 0.990 (0.981; 0.998) |
| DiasBP rest (mmHg) | 171 | 1.020 (0.999; 1.041) |
| DiasBP max (mmHg) | 181 | 1.002 (0.988; 1.016) |

Hazard ratios derived from Cox regression adjusted for age, sex and body mass index. *$p < 0.05$. PAOD: peripheral arterial occlusive disease; COPD: chronic obstructive pulmonary disease; LVEF: left vetricuar ejection fraction (%); TAPSE: tricuspid annular plane systolic excursion (mm), TI: tricuspid insufficiency; RAP: right arterial pressure; PAP: pulmonary artery pressure (mmHg); PAWP: pulmonary artery wedge pressure (mmHg); PVR: pulmonal vascular resistance (WU); CO: cardiac output (l); CI: cardiac index (l/min/m²); TLC: total lung capacity (l); VC: vital capacity (l); FVC: forced vital capacity (l); FEV1: forced expiratory volume in 1 second (l); RV: residual volume (l); DLCO: diffusion capacity (mmol/min/kPa); KCO: global diffusion capacity (mmol/min/kPa/l); $VO_2$: oxygen uptake (ml); AT: anaerobic threshold; HR: heart rate (bpm); $VO_2$/HR: oxygen pulse (ml/beat); VÉ/$VCO_2$: breathing efficacy; pet$CO_2$: end tidal carbon dioxide (mmHg); Aa$DO_2$: alveolar arterial oxygen difference (mmHg); Paet$CO_2$: gradient between pet$CO_2$ and arterial $CO_2$ levels (mmHg); VÉ/MVV: minute ventilation/maximum voluntary ventilation (%); IC: inspiratory capacity (l); EELV: endexpiratory lung volume (l); BF: breathing frequency (/min); VÉ: minute ventilation (l/min); VT: breathing volume (l); sysBP: systolic blood pressure (mmHg); diasBP: diastolic blood pressure (mmHg).
