# Supplementary material for: Value of Cardiopulmonary Exercise Testing in Prognostic Assessment of Patients with Interstitial Lung Diseases
Source: J Clin Med. 2022 Mar 14;11(6):1609. doi: 10.3390/jcm11061609 (PMC8954900; doi:10.3390/jcm11061609)
Supplement: Supplementary file 1 [file jcm-11-01609-s001.zip › Supplement table 3_JCM.pdf]

Table S3. Sensitivity, specificity, and Youden index for the perfect cut-offs for mortality (selected data).

Biomarker	Cut-Off	Youden Index	Sensitivity	Specificity
**FEV-1 (l)**				
All	96.7	0.19	43%	80%
IPF	92.8	0.31	48%	76%
**DLCO (mmol/min/kPa)**				
All	35.7	0.36	55%	79%
IPF	42.1	0.50	57%	88%
**KCO (mmol/min/kPa/l)**				
All	47.5	0.39	67%	76%
IPF	69.8	0.41	48%	90%
**VO_2 peak (ml/min/kg)**				
All	61.1	0.39	57%	80%
IPF	68.8	0.35	53%	80%
**VÉ/VCO_2 @ AT**				
All	39.0	0.30	50%	79%
PF	37.0	0.34	51%	88%
**petCO_2 peak (mmHg)**				
All	24.4	0.30	57%	74%
IPF	24.8	0.26	56%	71%
**VÉ/VCO_2 slope**				
All	40.4	0.34	56%	77%
IPF	32.8	0.32	48%	100%
**VO_2 peak/HR max (ml/beat)**				
All	8.61	0.38	61%	76%
IPF	12.96	0.23	45%	85%

IPF: idiopathic pulmonary fibrosis; FEV1: forced expiratory volume in 1 second (l); DLCO: diffusion capacity (mmol/min/kPa); KCO: global diffusion capacity (mmol/min/kPa/l); VO_2: oxygen uptake (ml); AT: anaerobic threshold; VÉ/VCO_2: breathing efficacy; petCO_2: end tidal carbon dioxide (mmHg); VO_2/HR: oxygen pulse (ml/beat).
